# Evidence for an Evolutionary Continuity in Social Dominance: Insights from Nonhuman Primate Tractography

**DOI:** 10.1523/JNEUROSCI.1646-24.2025

**Published:** 2025-07-21

**Authors:** Julie Royo, Thomas Orset, Marco Catani, Pierre Pouget, Michel Thiebaut de Schotten

**Affiliations:** ^1^Brain Connectivity and Behaviour Laboratory, Paris 75013, France; ^2^Sorbonne University, Inserm U1127, CNRS UMR7225, UM75, ICM, Movement Investigation and Therapeutics Team, Paris 75013, France; ^3^Department of Neuroscience, Imaging and Clinical Sciences, University “G. d’Annunzio” - Chieti-Pescara 66100, Italy; ^4^ITAB - Institute of Advanced Biomedical Technologies, University “G. d’Annunzio” - Chieti-Pescara 66100, Italy; ^5^Groupe d’Imagerie Neurofonctionnelle, Institut des Maladies Neurodégénératives-UMR 5293, CNRS, CEA University of Bordeaux, Bordeaux 33000, France

**Keywords:** behavior, imaging, limbic system, primates, social dominance, tractography

## Abstract

The dynamics of social dominance play a significant role in regulating access to resources and influencing reproductive success and survival in nonhuman primates. These dynamics are based on aggressive and submissive interactions that create distinct, hierarchically organized social structures. In humans, whose social behavior is similarly organized, the use of brain imaging based on tractography has identified key neuronal networks of the limbic system underlying social behavior. Among them, the uncinate fasciculus and the cingulum bundle have been associated with aggression and some disorders such as psychopathy. In this study, we have used advanced tractography to study the anatomy of connections underlying social dominance in a colony of 15 female squirrel monkeys (*Saimiri sciureus*). We correlated the biostructural properties of the uncinate fasciculus and cingulum with behavioral hierarchy measures while controlling for factors such as age, weight, handedness, brain size, and hormonal influences. The fornix, a limbic connection involved in memory, was also included as the control tract. Our findings indicate a significant correlation between the integrity of the right uncinate fasciculus and social dominance measures, including normalized David's scores, aggressive behaviors, and submissive behaviors. Trends observed in the left uncinate fasciculus hint at potential bilateral involvement with a right hemispheric lateralization. These results are consistent with human studies linking the uncinate fasciculus to social aggression and disorders, suggesting an evolutionary continuity in the neuroanatomical substrates of social dominance back to at least 35 million years.

## Significance Statement

In nonhuman primates, social dominance determines resource access and impacts survival and reproduction. In this study, we used advanced tractography to study the anatomy of connections related to social dominance focusing on limbic regions such as the uncinate fasciculus and cingulum. We found a strong correlation between the integrity of the right uncinate fasciculus and social dominance measures including hierarchy, aggressive, and submissive behaviors. These results suggest an evolutionary continuity in the neuroanatomical substrates of social dominance back to at least 35 million years.

## Introduction

Social dominance plays a crucial role in many animal species, especially nonhuman primates. Beyond theory, it regulates access to food, mates, and shelter (Moscovici, 1972; Smuts et al., 1987; De Waal and Luttrell, 1989; Klass and Cords, 2015; Tibbetts et al., 2022), and influences reproduction, grooming, hunting, and predator avoidance (Maslow, 1936; Varley and Symmes, 1966; Moscovici, 1972; Abbott, 1984; Digby, 1995). These hierarchies emerge from repeated interactions involving aggression and submission. Dominance establishes rank, while subordinate behaviors promote group stability. Similar layered structures are seen in human societies, shaped by interactions and social cues (Moscovici, 1972; Milewski et al., 2022; Tibbetts et al., 2022).

Diversity exists among societies of nonhuman primates (Moscovici, 1972; Swedell, 2012). Orangutans are mostly solitary; gibbons and some marmosets form monogamous pairs. Polyandrous groups, like in marmosets and tamarins, involve cooperative breeding. In polygynous groups such as gorillas, dominant males defend several females, often resulting in infanticide after takeovers. Multimale multifemale groups, seen in squirrel monkeys, baboons and macaques, are more complex, with philopatric females and dispersing males. Fission-fusion societies, like in chimpanzees and bonobos, form flexible subgroups depending on resources, while multi-level societies, like in hamadryas baboons, feature nested hierarchies (Swedell, 2012; Tibbetts et al., 2022). Squirrel monkeys (Saimiri), exhibit social flexibility shaped by species, sex and context (Mulholland et al., 2020). In Saimiri boliviensi, male status correlates with testosterone, while female order is variable (Mendoza et al., 1978; Williams and Glasgow, 2000). Saimiri sciureus shows male hierarchies with low competition but high resource rivalry (Mitchell et al., 1991). In Saimiri oerstedi, males monitor female fertility without rigid rank (Mitchell et al., 1991). Saimiri collinsi shows size-based aggression (Pinheiro and Lopes, 2018). Group composition affects dynamics: male introductions can reduce female aggression and shift alliances (Anschel and Talmage-Riggs, 1977; Wooley et al., 1978). These adaptable hierarchies impact social interactions and resource access (Mendoza et al., 1978; Mitchell et al., 1991). Our study focused on Saimiri sciureus, whose social hierarchy may relate to specific brain networks.

Brain evolution is tied to increases in brain size (Zhang and Sejnowski, 2000; Herculano-Houzel et al., 2010; van Essen, 2018), affecting white matter fiber length (Kaas, 2000). Larger brains require longer fibres, slowing information transfer. Myelination partly compensates, but spatial and metabolic constraints limit efficiency (Ringo et al., 1994). These trade-offs may reflect evolutionary adaptations. Diffusion magnetic resonance imaging (MRI) tractography enables cross-species comparisons of white matter architecture (Friedrich et al., 2021; Yokoyama et al., 2021; Yang et al., 2022; Assimopoulos et al., 2024, 2025).

In humans, white matter tracts like the uncinate fasciculus and cingulum relate to social behavior. The uncinate fasciculus connects frontal and temporal limbic regions, regulating socially (Coad et al., 2020). Its dysfunction has been associated with antisocial behavior, including in frontotemporal dementia (Bang et al., 2015), brain lesions (Grafman et al., 1996; Thiebaut De Schotten et al., 2015), and psychopathy (Craig et al., 2009). More broadly, the cingulum, connecting parts of the limbic system (Alves et al., 2019), plays a role in mental states (Catani et al., 2013) and emotional regulation (Sethi et al., 2015).

Although dominance and psychopathy are strongly correlated in humans (Kosson et al., 1997; Ross et al., 2009; Rauthmann and Kolar, 2013), studies in nonhuman primates remain limited. In macaques, findings link rank with activity in amygdala, striatum and frontotemporal networks (Noonan et al., 2014), but small cohorts limited generalization. Studying more distantly related primates can help clarify the evolutionary roots of dominance.

This study examined three key fiber tracts—the uncinate fasciculus, cingulum, and fornix—in squirrel monkeys to assess their relationship with social hierarchy. Our analysis included 15 female squirrel monkeys and controlled for variables like age, weight, handedness, brain size, and sexual hormones. Our goal is to identify neural features linked to dominance and test whether primate brain systems involved in social rank mirror those in humans, offering insight into neurological and psychiatric conditions.

## Materials and Methods

### Animals and housing conditions

All data were acquired from 15 female squirrel monkeys (*Saimiri sciureus*) born in captivity at the Primatology Station of the CNRS (UPS846 CNRS, Agreement C130877). Animals live in a social group within Paris Brain Institute (ICM), housed in a multicompartment cage complex (17.5 m^3^), which can be divided into two 8.75 m^3^ enclosures, with structural and manipulable enrichments (ladders, platforms, hammock, etc.) at constant temperature (24–26°C), relative humidity (55%), and a 12 h light/dark cycle. The animals had *ad libitum* access to water, and food was given three times a day, including commercial monkey chow, fruits, vegetables, and other various enrichments (eggs, nuts, seeds, mealworms, etc.). During the hierarchy test, animals were between 4 and 9 years old and weighed between 660 and 825 g. All imaging data were obtained 1 year later (ages between 5 and 10 years old and weighing between 650 and 800 g with a maximum variation between −85 and +50 g compared with behavioral tests; Table 1).

**Table 1.** Hormonal values, mean age, and weight of each animal during the behavioral test and MRI scans

ID	Testosterone (absorbance)	Progesterone (absorbance)	Mean age (years)	Mean weight (grams)
*MAR*	1.201	0.244	6.5	712.5
*COR*	0.932	0.205	5.5	805.0
*JYN*	1.071	0.273	4.5	687.5
*KAN*	1.155	0.308	7.5	775.0
*ROS*	1.208	0.317	8.5	730.0
*AGI*	0.774	0.319	6.5	720.0
*MAZ*	0.827	0.300	5.5	800.0
*WYN*	1.217	0.221	6.5	677.5
*MON*	1.048	0.199	4.5	675.0
*IYE*	0.719	0.250	7.5	775.0
*ENA*	1.157	0.354	5.5	737.5
*PYT*	1.083	0.268	7.5	717.5
*GAI*	0.968	0.285	7.5	752.5
*ASA*	1.069	0.248	8.5	667.5
*GAL*	0.774	0.319	9.5	767.5

Hair samples for hormonal analysis were collected from all animals on the same day during the monthly weighing session. Fifteen animals were weighed once a month during the behavioral testing period, while for the day of the MRI scans.

All procedures were designed in association with the veterinarian team of the ICM (agreement number B75-13-19), approved by the Regional Ethical Committee for Animal Experiments (Charles Darwin CE005 under the project reference APAFIS #21086-2019061415485300).

### Behavioral test: hierarchy/dominance

**Figure 1.**
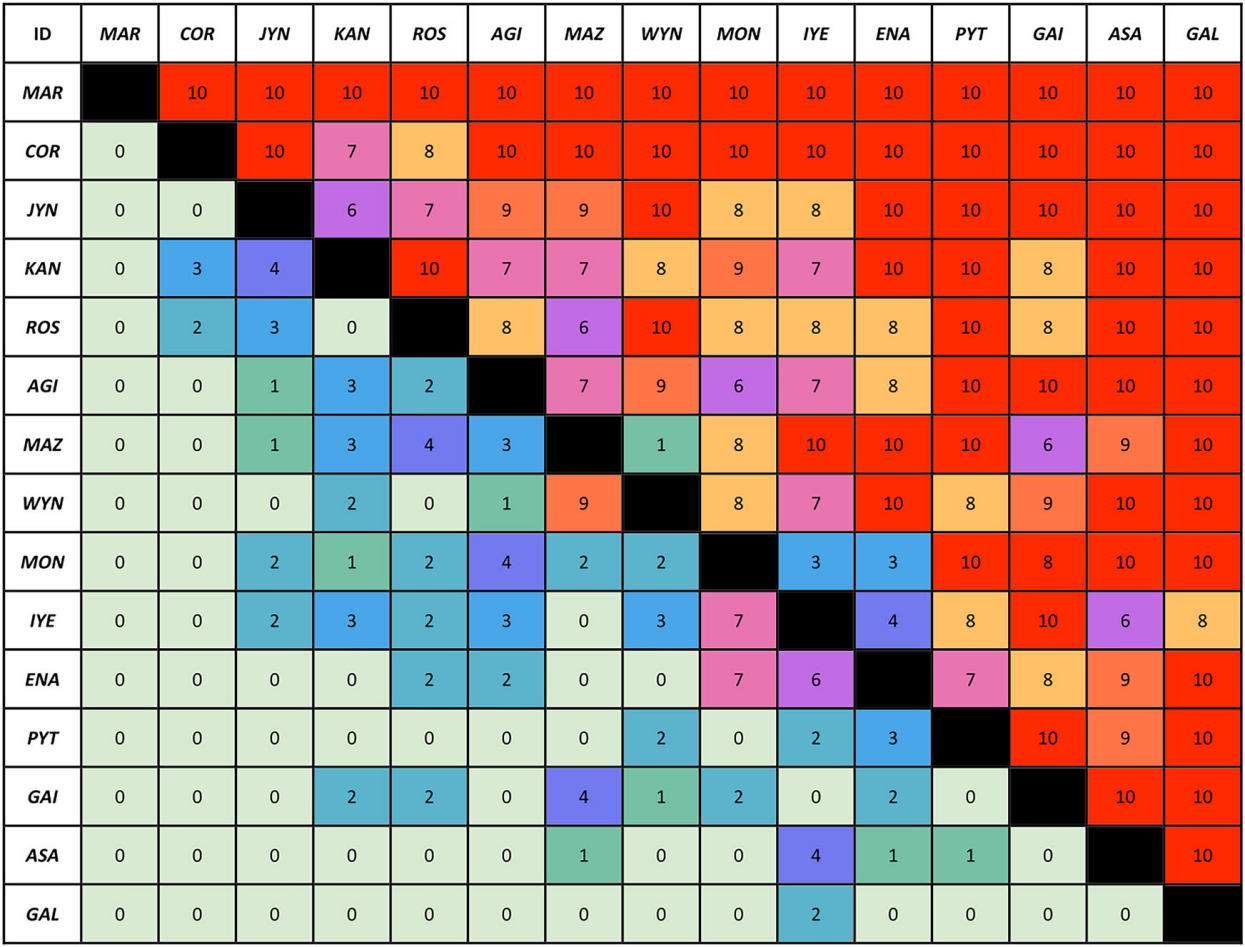
Pairwise interactions. Each row represents the total number of victories achieved by the animal listed in the row against the animal specified in the column. The cells are color-coded based on the number of victories, ranging from green (0 victories) to red (10 victories). The dataset includes 15 animals, with a total of 105 pairwise interactions (14 interactions per animal).

Each pair of animals participated in behavioral tests, which was conducted in one side of their housing cage (∼8.75 m^3^), ensuring a focused and controlled environment that maintained visual, olfactory, and auditory contact with the rest of the group, which was crucial for accurate results. A selection phase for rewards was conducted, and dried grapes were found to be the preferred choice for all animals. The primary test consisted of a food presentation test conducted in the morning, just before the daily food distribution. To ensure fairness, the experimenter delayed the start of each trial until both animals were positioned equidistantly from the grape at the opposite end of the cage and oriented toward the reward. Each pair underwent 10 trials, during which a range of behaviors were recorded. Two broad categories of behaviors have been defined: aggressive and submissive behaviors. Aggressive behaviors included actions such as fighting, making threats, vocalizations, pursuits of others, and food thefts often aimed at establishing social hierarchy or securing resources. In contrast, submissive behaviors involved retreating, avoiding confrontation or adopting submissive postures, and vocalizations to evade aggressive encounters. Each trial lasted ∼2 min, ending when the winning animal finished consuming the reward. The results, including wins and losses, were compiled for each social pairing. Over 26 d of testing, each animal was paired with another animal once, resulting in 105 unique pairs (Fig. 1). The pairing and testing schedule was randomized, with the restriction that no animal was tested consecutively, ensuring a preference for only one test per day. In cases where an animal was tested twice in 1 d, a minimum rest period of 20 min was enforced between tests. Every test was conducted by a dedicated team comprising two researchers: an experimenter, who presented the rewards to the animals, and an independent observer, who meticulously recorded and classified the behaviors in real time. All observations followed a standardized protocol to ensure consistency across trials and animals. This collaborative approach ensures a comprehensive understanding and consistent analysis of the animals’ responses, enhancing the reliability of our findings. To ensure the stability of the social hierarchy, the dominance test was repeated 1 year later, after the MRI scan, revealing no significant changes in the hierarchy structure.

### Hormonal analysis

Measurement of sexual hormones, specifically testosterone and progesterone, was conducted following the protocol established by Koren et al. (2002), 8 months after the behavioral test (hormonal values in Table 1). While testosterone and progesterone are known to be sensitive to short-term fluctuations associated with the estrous cycle in females, these fluctuations are often predictable and can be influenced by stable physiological factors. To ensure that hormone levels accurately reflected the individuals’ overall hormonal profiles, we carefully selected a period outside the animals’ reproductive cycle for sampling. Measuring sexual hormones outside the reproductive period provides a better understanding of long-term hormonal dynamics. With less pronounced fluctuations, these stable measurements make it possible to assess the impact on behavior and health more reliably.

During the manual restraint process, the belly hairs from each animal were shaved, collecting ∼50 mg per animal in a dry container. In the laboratory, the hairs were carefully weighted, washed with isopropanol, and cut into 3–4 mm pieces using fine scissors before being stored in an Eppendorf tube. Methanol was added to the Eppendorf tube, followed by sonication for 30 min. Subsequently, the tubes were incubated overnight at 50°C with gentle agitation. Methanol was removed with a pipette, and the rest was evaporated to dryness under a stream of nitrogen. Each sample was reconstituted with phosphate-buffered saline (PBS), pH 7.0, and dispensed into the appropriate polyclonal hormone antiserum-coated well of an ELISA (solid phase enzyme-linked immunoassay) microplate, in duplicates for testosterone (EA78 EIA buffer, Oxford Biomedical Research) and progesterone (EA74 EIA buffer, Oxford Biomedical Research) kit analysis. The microplate was incubated at room temperature for 5 min. Then, the enzyme-conjugate was added to each well, and the microplate was thoroughly mixed for 10 s. The microplate was incubated at room temperature for 60 min before washing it. After the washing step, the substrate solution was added and incubated for an additional 15 min at room temperature. To stop the enzymatic reaction, a stop solution (2N HCl) was added. The absorbance of each well was measured at 450 nm. The observed values were inversely proportional to the concentration of the hormone present. All standards and reagents were provided in the kits.

### Dominance data analysis

All data were analyzed with R software (4.3.0).

Based on the win/loss ratios for all pairwise interactions performed by each animal (Fig. 1), we analyzed the results using David's score to estimate the hierarchical rank of the animals (David, 1987). David's score is an appropriate approach for ranking animals from highest to lowest by considering repeated interactions within the group and employing rank-sum scoring. This method combines unweighted and weighted sums of dyadic win and loss proportions, factoring in each individual's overall success. This means that defeating a high-ranking opponent carries more weight than defeating a low-ranking one, allowing for a more nuanced assessment of success. Each dyad is assessed based on an equal number of interactions to maintain fairness. Although agonistic behaviors such as aggression and submission may influence the outcome of interactions, they do not directly enter into the computation of David's score. Rather, the score is solely based on the outcomes—wins and losses—which may themselves result from these behaviors. Thus, the method provides a behavioral-independent measure of social rank while still reflecting the consequences of individual strategies during confrontations. The animal with the highest score was identified as dominant, the one with the lowest score as subordinate, and the animal with the middle score as subdominant. The stability of social dominance was assessed by calculating social dominance status based on David's scores 1 year later. Dominance ranks are determined by assigning wins and losses in individual dyadic agonistic interactions. Simple ordinal ranks are generated by numbering individuals according to their position in the matrix (1, 2,…*n*, where *n* represents the size of the hierarchy). The highest-ranking animal was assigned by 1, and the lowest was given by *n*. David's scores are normalized to ensure that all individuals’ scores range from 0 to *n*−1:

$$\mathrm{Davi}d^{\prime }s\;\mathrm{score}=w+w2-l-l2,$$

where *w* denotes sum of win values, *w*2 signifies the sum of weighted *w* values, *l* refers to the sum of loss values, and *l*2 stands for the sum of weighted *l* values. Steepness method measures the clarity of this hierarchy, with high steepness reflecting a clear, rigid structure and low steepness suggesting a more flexible, egalitarian social order. These methods help describe both individual rank and overall social structure within a group. Analyses were performed with the “steepness” library (version 0.3-0: https://cran.r-project.org/web/packages/steepness/index.html).

### MRI

#### Anaesthesia

Anesthesia was induced with an intramuscular (IM) injection of alfaxalone (3 mg/kg), which was then maintained with isoflurane (1.5–3%) during animal preparation. During the MRI session, the animal was kept sedated by administering an IM injection of Dexdomitor (0.5 mg/kg) and maintaining anaesthesia with perfusion of alfaxalone (12 mg/kg). Respiratory rate and internal temperature were monitored during MRI acquisition. To prevent awakening, an increase in alfaxalone's flow rate was decided after 2 h in the MRI scanner or if the respiratory rate rose over 60/min (18 mg/kg). A warm water flow maintained body temperature between 34.5 and 38.5°C. Anesthesia was sustained for 6 ± 0.5 h from induction to awakening.

#### Acquisition parameters

The necessary imaging data for this study is available in Figshare (Orset et al., 2023a).

Magnetic resonance images were acquired using a Biospec USR 117/16 (Bruker). Radiofrequency emission and signal reception were performed using a 72 mm birdcage transceiver (Bruker). An automatic shim was computed using the MAPSHIM routine on an ellipsoid that fits the whole squirrel monkey brain before image acquisition. The field map obtained after shimming was estimated at 72 × 72 × 72 mm^3^ to perform later distortion correction. Diffusion-weighted images were then acquired using a fat-saturated 3D-DW-segmented EPI sequence with the following parameters: four segments, TR/TE = 200/24 ms, bandwidth = 600 kHz, Mtx = 160 × 160 × 128, and FOV = 64.0 × 64.0 × 51.2 mm^3^, leading to an isotropic resolution of 400 × 400 × 400 µm^3^. Diffusion gradient duration *δ* = 3 ms and gradient separation Δ = 11 ms. One hundred noncollinear diffusion gradient directions were acquired using *b*-values: 2,000, 1,000, and 300 s/mm^2^ with 64, 29, and 7 directions, respectively. Directions were generated offline on a sphere using Caruyer's online Q-space sampling tool (Caruyer et al., 2013). Additionally, eight nondiffusion weighted volumes were acquired. It should be noted that the entire scanning process for this multishelled diffusion acquisition took 3 h and 4 min per animal.

#### Preprocessing

The raw data were denoised using the *denoise* function from MRtrix3 and reoriented using ExploreDTI (www.exploredti.com), which then concatenated the three *b*-values (Orset et al., 2023b). Next, a brain mask was generated and manually corrected with the assistance of BrainSuite21a (www.brainsuite.org). This involved extending the mask to missing areas (due to a signal drop) and eroding the masked cerebrospinal fluid. To ensure accuracy, any distortions caused by eddy currents and movement were corrected using the eddy tool from FSL.

#### Tractography

The multishell diffusion-weighted images were analyzed using deterministic tractography analysis, which was performed by StarTrack (http://www.mr-startrack.com) in conjunction with MATLAB, utilizing the damped Richardson–Lucy spherical deconvolution algorithm (Dell’Acqua et al., 2013; Guo et al., 2020; Orset et al., 2023b). The parameters for spherical deconvolution were set to ALFA = 2, iterations = 1,000, *n* = 0.001, and *r* = 8. Whole brain deterministic tractography was carried out via two runs of the M-Euler algorithm, with rigorous parameters such as an absolute threshold of 0.005, a step size of 0.4 mm, an angle threshold of 35°, and minimum and maximum streamline lengths of 8 and 100 mm, respectively.

#### Dissections

Manual tractography dissections of the uncinate fasciculus, the fornix and the cingulum were performed using TrackVis (www.trackvis.org) by the expert anatomist M.T.d.S.

### Statistical analysis

All data were analyzed with R (version 4.3.0). We extracted some parameters from the data imaging: total brain volume and hindrance modulated orientational anisotropy (HMOA) for the uncinate fasciculus, the fornix, and the cingulum. HMOA is a fiber-specific diffusion index enhancing the ability to capture the directionality and complexity of neural tracts, particularly in areas characterized by crossing fibers. HMOA detects subtle changes in axonal integrity from microstructural alterations, providing precise fiber orientation information (Dell’Acqua et al., 2013; Rojkova et al., 2016). A Shapiro–Wilk test was used to test the normality of the data. Pearson’s correlations were performed in JASP (https://jasp-stats.org). A multiple linear regression model was performed to determine how total aggressive and submissive behaviors (Table 2) might influence the relationship between social dominance scores and white matter integrity. The model was adjusted for these behavioral variables and evaluated regression coefficients to assess their impact. The results were analyzed using *p*-values and standardized regression coefficients, with a significance level set at 0.05.

**Table 2.** Observed behaviors, rank, and normalized David's score

ID	Rank	Normalized David's score	Total wins (%)	Threats (%)	Vocalizations (%)	Fights (%)	Pursuits (%)	Thefts (%)	Total of aggressive behaviors (%)	Withdrawal (%)	Theft victim (%)	Total of submissive behaviors (%)
*MAR*	1	14.0	100.00	35.71	35.71	14.29	0.00	0.00	17.14	0.00	0.00	0.00
*COR*	2	12.5	89.29	35.71	42.86	7.14	0.00	0.00	17.14	7.14	0.00	3.57
*JYN*	3	10.7	76.43	0.00	0.00	7.14	0.00	0.00	1.43	14.29	0.00	7.14
*KAN*	4	10.3	73.57	35.71	35.71	7.14	0.00	14.29	18.57	21.43	0.00	10.71
*ROS*	5	9.1	65.00	0.00	0.00	0.00	0.00	0.00	0.00	28.57	7.14	17.86
*AGI*	6	8.3	59.29	7.14	7.14	7.14	0.00	0.00	4.29	28.57	0.00	14.29
*MAZ*	7	7.5	53.57	21.43	21.43	7.14	0.00	0.00	10.00	50.00	0.00	25.00
*WYN*	8	7.4	52.86	0.00	7.14	0.00	0.00	0.00	1.43	50.00	0.00	25.00
*MON*	9	5.7	40.71	0.00	0.00	7.14	0.00	0.00	1.43	64.29	0.00	32.14
*IYE*	10	5.6	40.00	0.00	0.00	0.00	0.00	0.00	0.00	50.00	0.00	25.00
*ENA*	11	5.1	36.43	0.00	0.00	0.00	0.00	0.00	0.00	57.14	0.00	28.57
*PYT*	12	3.6	25.71	0.00	0.00	0.00	0.00	0.00	0.00	78.57	0.00	39.29
*GAI*	13	3.3	23.57	0.00	0.00	0.00	0.00	0.00	0.00	85.71	0.00	42.86
*ASA*	14	1.7	12.14	0.00	0.00	0.00	0.00	7.14	0.00	92.86	0.00	46.43
*GAL*	15	0.02	1.43	0.00	0.00	0.00	0.00	0.00	0.00	92.86	14.29	53.57

Behaviors were classified as positive outcomes when a minimum of one specified behavior was observed during interactions involving a dyad. In instances where no specific behaviors were recorded, a neutral outcome was documented. Results were then assigned to the respective animal involved in each interaction.

## Results

**Figure 2.**
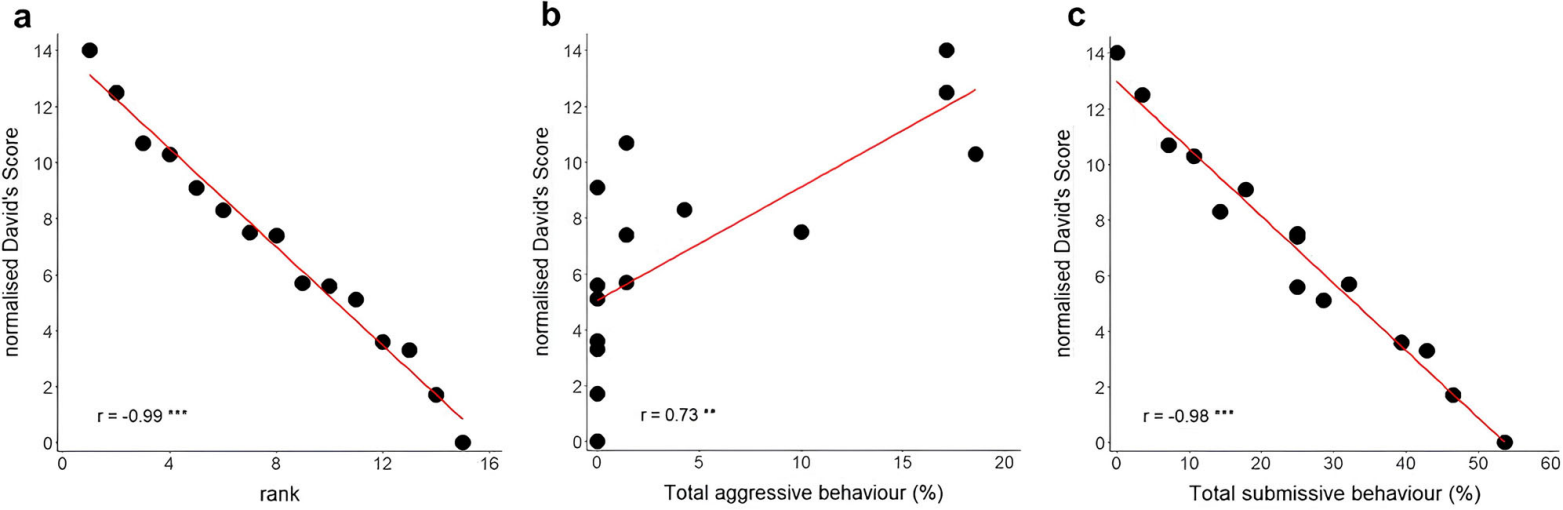
Measure of the hierarchy within a nonhuman group. Normalized David's scores plotted against (***a***) rank order, (***b***) aggressive, and (***c***) submissive behavior. ***p < 0.001.

The proportion of win–loss pairwise interactions between individuals in a group (i.e., normalized David's score) typically defines their hierarchy and was particularly steep in our colony of squirrel monkeys (Fig. 2*a*) in comparison with other large colonies of species such as lemurs (Norscia and Palagi, 2015), black-and-white snub-nosed monkeys (Cui et al., 2014), or also macaques (Balasubramaniam et al., 2013). Additionally, dominance can be further characterized through the quantification of aggressive and submissive behaviors both demonstrating a strong correlation with the normalized David's score (Fig. 2*b,c*; *r* = 0.750, *p* < 0.001 and *r* = −0.976, *p* < 0.001, respectively). Classical factors including weight, handedness, brain size, and hormonal factors (testosterone and progesterone) did not influence these measures significantly (all *p* > 0.05). Importantly, age demonstrated a trend of influence on the normalized David's score (*r* = −0.502, *p* = 0.056) and was therefore covariated out of subsequent analyses. We also added brain sizes as a covariate to control for its potential influence on white matter neuroanatomical measurements.

**Figure 3.**
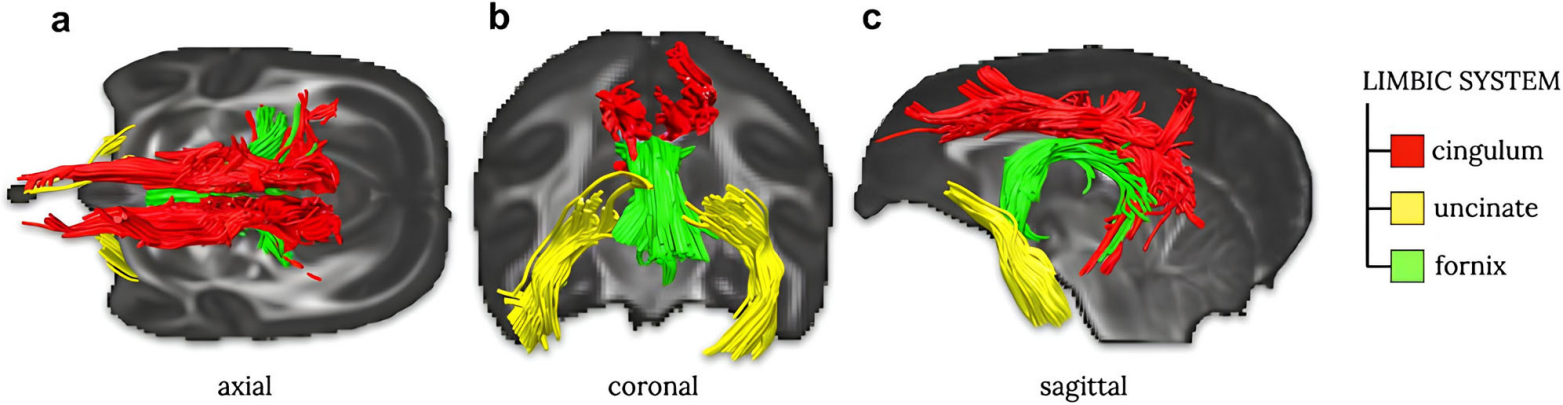
Tractography of the limbic system in (***a***) axial, (***b***) coronal, and (***c***) sagittal view. Cingulum fibers have been colored in red, uncinate in yellow, and fornix in green.

Advanced tractography (Fig. 3) derived from multishell high-resolution diffusion-weighted imaging was employed to extract the uncinate fasciculus and the cingulum tract in both hemispheres together with the fornix and collect their integrity [i.e., orientational anisotropy (Dell’Acqua et al., 2013)].

**Figure 4.**
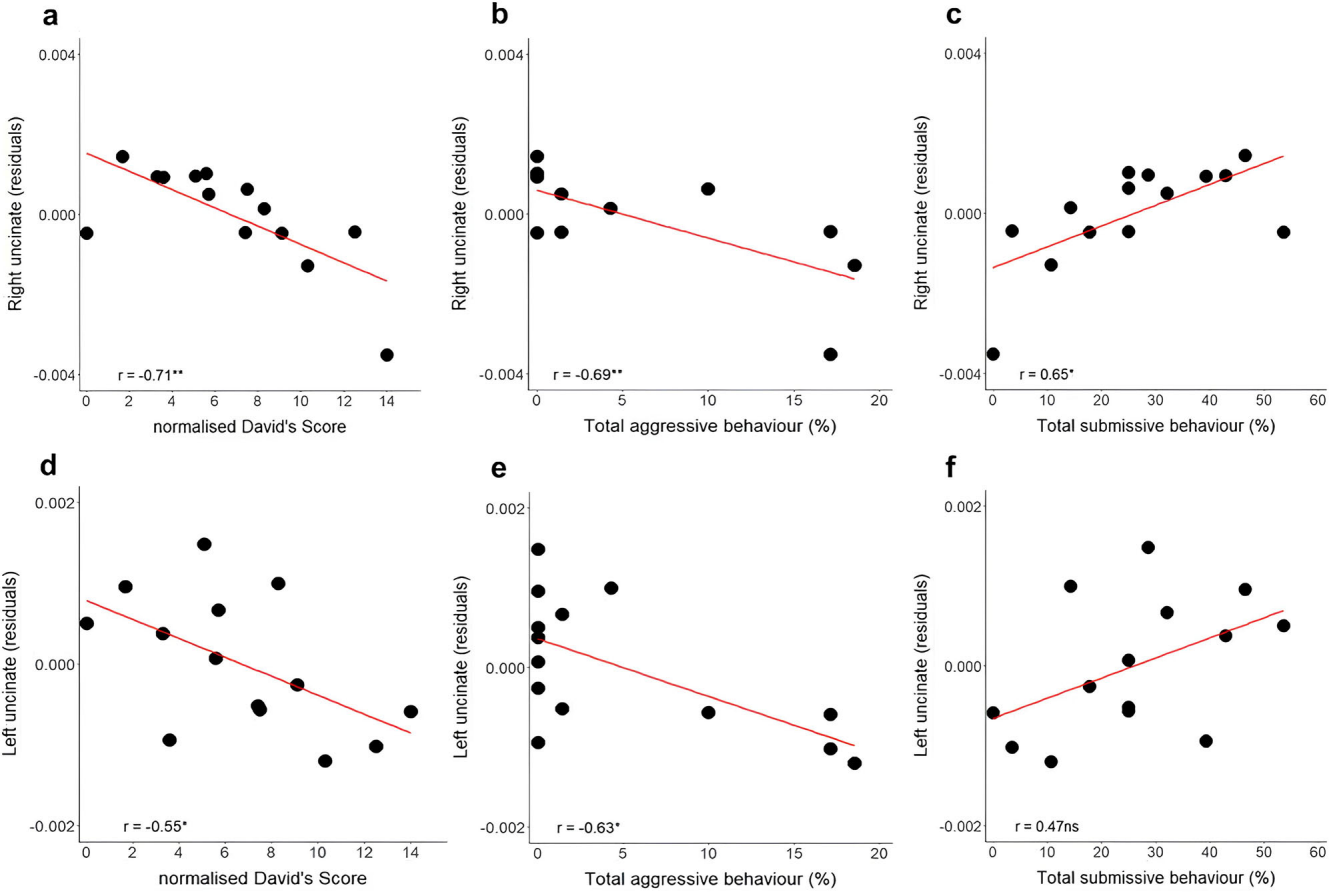
Relationship between the right (***a–c***) and left (***d–f***) uncinate fasciculi and dominant behavior quantified as normalized David's score (***a***, ***d***), aggressive behavior (***b***, ***e***), and submissive behavior (***c***, ***f***). **p < 0.01 *p < 0.05. Note that *p* < 0.01 is significant after correction for multiple comparisons. One primate was excluded due to the lack of exploitable neuroimaging data. Residuals correspond to orientational anisotropy values covariated out for brain size and age.

Results indicated that the integrity of the right uncinate fasciculus correlated significantly with the normalized David's score (*r* = −0.71, *p* = 0.004) and aggressive (*r* = −0.69, *p* = 0.006) and submissive (*r* = 0.65, *p* = 0.012) behaviors (Fig. 4*a–c*, respectively). Importantly, the left uncinate fasciculus also displayed some trend of correlations (normalized David's score, *r* = −0.55, *p* = 0.042; aggressive behavior, *r* = −0.63, *p* = 0.016; and submissive behavior, *r* = 0.47, *p* = 0.087) that did not survive Bonferroni’s correction for multiple comparisons (set at *p* < 0.01 for five tract comparisons; Fig. 4*d–f*).

Cingulate integrity, evaluated on both the left and right sides, demonstrated no significant correlation with normalized David's score (left, *r* = −0.40, *p* = 0.155; right, *r* = −0.43, *p* = 0.121). Additionally, there were no significant correlations with aggressive behavior (left, *r* = −0.27, *p* = 0.344; right, *r* = −0.36, *p* = 0.209) or submissive behavior (left, *r* = 0.38, *p* = 0.184; right, *r* = 0.36, *p* = 0.207). Likewise, the fornix did not exhibit significant correlations with normalized David's score (*r* = −0.33, *p* = 0.245), aggressive behavior (*r* = −0.12, *p* = 0.686), or submissive behavior (*r* = 0.36, *p* = 0.211).

To further investigate the relative contributions of aggressive and submissive behaviors to the relationship between social dominance scores and white matter integrity, we performed a regression model. Specifically, we explored whether the correlation between white matter integrity (right uncinate fasciculus) and social dominance scores persisted after controlling for aggressive and submissive behaviors. The results revealed that when aggressive and submissive behaviors were included as covariates, the statistical relationship between social dominance scores and white matter integrity disappeared (all *p* > 0.15). The residual values, accounting for 3% of the variance in social dominance, could not be explained by white matter integrity. This suggests that the previously observed relationship is largely driven by the behavioral components (aggressive and submissive behaviors) rather than the social dominance scores themselves.

## Discussion

These results suggest a significant role of specific white matter tracts of the limbic system in mediating social dominance behaviors in squirrel monkeys, adding to prior primate studies (Koski et al., 2015; Santamaría-García et al., 2015; Ferreira-Fernandes and Peça, 2022; Tibbetts et al., 2022). These findings expand our understanding of the evolutionary origins of social behavior and its neuroanatomical foundations.

Our colony exhibited a pronounced social hierarchy, indicated by the steep normalized David's score (David, 1987; Gammell et al., 2003; Balasubramaniam et al., 2013), reinforcing earlier reports on squirrel monkey social structure (Mendoza et al., 1978; Mitchell et al., 1991). Compared with other primate groups such as lemurs (Kaburu and Newton-Fisher, 2015; Butovskaya, 2020; McCowan et al., 2022; Neumann and Fischer, 2023), the steepness value (0.8729) indicates a highly despotic social structure, surpassing even chimpanzees (0.22–0.70; Hayaki et al., 1989; Kaburu and Newton-Fisher, 2015). Steepness, quantifying hierarchies from 0 (egalitarian) to 1 (despotic), reveals dominance dynamics where high-ranking individuals monopolize resources, limiting subordinates. This pattern supports theories proposing that subordinates engage in grooming or affiliative behaviors to gain advantages. Interestingly, traditional factors such as body mass, manual dominance, encephalization, and endocrinological variables had little influence, highlighting the importance of social interactions in shaping dominance. These findings underscore the role of limbic circuits in social dynamics (Catani et al., 2013; Alves et al., 2019). Importantly, David's score quantifies dominance through the outcomes of dyadic encounters—wins and losses—rather than through the aggressive and submissive behaviors themselves. Although such behaviors may influence these outcomes, they are not directly integrated into the calculation of the score. Instead, the score reflects the cumulative success of individuals in social contests, allowing for a behaviorally neutral yet outcome-sensitive assessment of dominance. This property makes it a particularly robust measure when investigating the neurobiological underpinnings of social hierarchy, as it anchors dominance in interaction outcomes rather than subjective behavioral classifications.

Our tractography analysis revealed a significant correlation between orientational anisotropy of the right uncinate fasciculus and social dominance. Lower integrity was associated with higher David's scores, increased aggression, and reduced submissive behavior, suggesting a role in facilitation dominance-related behaviors. However, this relationship seems driven, in part, by the underlying aggressive and submissive behaviors themselves, rather than a direct link to broader social dominance. Although not surviving Bonferroni’s correction, the trends in the left uncinate fasciculus suggest potential bilateral involvement, with stronger influence from the right hemisphere. This observation aligns with theories of right hemispheric predominance in emotional processing in humans (Silberman and Weingartner, 1986; Demaree et al., 2005; Ioannucci et al., 2020). However, given the correlative nature of our analysis, it is unclear whether or not biological changes in the uncinate fasciculus were driving the excessive aggression or whether they are secondary to social dominance experience. Higher orientational anisotropy may reflect increased myelination or axonal cohesiveness, possibly as a response to social experience. Structural and functional connectivities are linked, with increased cortical activation from task-specific learning promoting oligodendrocyte activation and myelination along active cortical areas (McKenzie et al., 2014).

The uncinate connects the orbitofrontal cortex to the amygdala, involved in fear conditioning, a form of emotional learning where a neutral stimulus elicits defensive behavior after pairing with an aversive event (LeDoux, 1996). In humans, orbitofrontal cortex–amygdala connectivity, mediated by the uncinate fasciculus, predicts social anxiety (Phan et al., 2006). Our results may suggest that individuals exposed to aggressive social dynamics experience more pressure, embarrassment, and frustration requiring the orbitofrontal cortex to regulate the amygdala's response to negative emotions. This interpretation indicates that the relationship between social dominance and white matter integrity may be mediated by aggression and submission, rather than a direct effect of social dominance. Despite correlations between uncinate fasciculus integrity and social dominance behaviors, this relationship seems largely driven by aggression and submission, rather than a distinct dominance mechanism. Therefore, behaviors related to social dominance are not solely linked to a broad dominance construct but are intrinsically tied to specific aggressive and submissive behaviors.

Alternatively, white matter differences in the uncinate fasciculus may reflect different neurodevelopmental trajectories of limbic pathways that shape social aggression and dominance. Individuals with less myelinated uncinate fasciculus could be more prone to impulsive and aggressive responses in conflict situations. Aggression is crucial for survival and dominance, allowing humans and animals to secure resources and maintain hierarchies. However, excessive aggression, seen in certain neurological and psychiatric disorders such as dementia and psychopathy, can be maladaptive (Nelson and Trainor, 2007; Craig et al., 2009; Bang et al., 2015; Zhu et al., 2019). Our study focuses on nonpathological, socially relevant aggression within a stable hierarchy, providing insights into neural substrates of dominance-related behaviors. By connecting these findings to human conditions, we offer perspectives on the evolutionary origins of social behavior and brain mechanisms underlying dominance. The parallels between squirrel monkeys and humans suggest an evolutionary continuity in the neural substrates of social dominance (Adolphs and Birmingham, 2012; Kumaran et al., 2012; Chang et al., 2013; Watanabe and Yamamoto, 2015; Yokoyama et al., 2021; Oesch, 2024). Mechanisms supporting social dominance may have existed for at least 35 million years, dating back to our common ancestor with squirrel monkeys (Royo et al., 2021). These conclusions are supported by a recent tractography study reporting no differences between humans and Old World monkeys in the relative volume of the uncinate fasciculus (Barrett et al., 2020).

Our study's limitations include a small sample size and a focus on female squirrel monkeys, which may limit generalizability. Historically, neuroscience research has focused on male subjects, leading to female underrepresentation. Male mammals generally display higher aggression than females (Archer, 2009; Slater et al., 2009; Muller, 2010; Georgiev et al., 2013; Wranghama, 2017; Choleris et al., 2018; Sabbi et al., 2021; Evans et al., 2022). In contrast, females, including in humans, primates, and rodents, show stronger stress responses and defensive behaviors (Slater et al., 2009; Jolles et al., 2015; Choleris et al., 2018). Males compete for territory and resources, while females focus on food and reproduction. Examining sex differences in brain connectivity will improve understanding of behavioral disparity. Connectivity studies suggest that males exhibit stronger functional connectivity between cognitive and sensorimotor regions (Filippi et al., 2013), although some research shows reduced functional connectivity (İçer and Baş, 2020) or no significant differences (Weissman-Fogel et al., 2010). Sex differences have been identified in brain regions such as the cingulate cortex and limbic and prefrontal areas, supporting findings in marmosets (Nephew et al., 2020; Weis et al., 2020). Male marmosets have positive correlations between cognitive performance and activity in the dorsolateral prefrontal cortex and motor areas, along with significantly stronger neural connectivity compared with females. These findings suggest that differing connectivity patterns are critical in understanding cognitive variations between genders. Addressing this imbalance will improve our understanding of brain function and outcomes in medicine and psychology showing a more complete picture of sex differences. Future research should replicate these findings in larger and more diverse primate populations, including males. Social dominance measurements could be improved by analyzing group behaviors with more than two individuals and exploring different contexts. In addition, our observations also suggest subgroups within the cohort, and examining behaviors in these subgroups could yield important insights.

Future studies should explore other white matter tracts or brain regions to identify additional neural substrates for social behavior. Our analysis focused on basic emotions such as anger and fear, but more complex emotions, such as embarrassment or frustration, may influence social behaviors and group responses. Incorporating these emotions and their brain networks in future studies could deepen our understanding of the mechanisms behind social behavior. When analyzing aggressive and submissive behaviors in relation to David's score, it is essential to recognize their connection. The correlation between social behavior and the integrity of the uncinate needs careful interpretation. The relationship between the integrity of the uncinate and social dominance may be influenced by aggression (lead to victories and higher scores) and submission (result in defeats and lower scores), rather than a direct effect of social dominance. This suggests that the associations may reflect the impact of aggression and submission themselves, not a direct link between social dominance and white matter integrity. Further research should distinguish the effects of aggression and submission, as these behaviors likely influence brain regions differently, highlighting the complex relationship between social behavior and brain networks.

In conclusion, our results align with human studies linking the uncinate fasciculus to social behaviors characterized by antisocial behavior and aggression, suggesting an evolutionary continuity in the neural substrates of social dominance. By bridging findings from nonhuman primates to human conditions, our research offers insights into the evolution of social behavior and potential pathways for addressing aggressiveness and social disorders.
